# Evidence of Unhealthy Dietary Patterns in the School Lunch Sent from Home for Children in Mexico City

**DOI:** 10.3390/ijerph191811650

**Published:** 2022-09-15

**Authors:** Brenda Jazmín Flores-Moreno, Gloria Martínez-Andrade, Miguel Klünder-Klünder, América Liliana Miranda-Lora, Brenda Beristain-Lujano, Samuel Flores-Huerta, Eugenia Mendoza, Ariana Mayorga-Lima, Ximena Duque, Jenny Vilchis-Gil

**Affiliations:** 1 Epidemiological Research Unit in Endocrinology and Nutrition, Hospital Infantil de México Federico Gomez, Ministry of Health (SSA), Mexico City 06720, Mexico; 2 Academic Area of Nutrition, Institute of Health Sciences, Autonomous University of the State of Hidalgo, Pachuca Hidalgo 42000, Mexico; 3 Research Division, Mexico Children’s Hospital Federico Gómez, Mexico City 06720, Mexico; 4 Research Committee, Latin American Society for Pediatric Gastroenterology, Hepatology and Nutrition (LASPGHAN), Mexico City 06720, Mexico; 5 Infectious Diseases Research Unit, Pediatric Hospital, Mexican Institute of Social Security, Mexico City 06600, Mexico; 6 Medicine Faculty, National Autonomous University of Mexico, Mexico City 06320, Mexico

**Keywords:** children, school lunch, dietary patterns, nutritional status

## Abstract

The identification and characterization of dietary patterns are tools that are used to assess associations between diet and health or disease conditions. In Mexico, studies have examined dietary patterns in children for breakfast or for the whole day, but not specifically for their school lunch. The aim was to describe dietary patterns identified in school lunch and their association with the nutritional status and metabolic parameters of schoolchildren. In this cross-sectional study on schoolchildren from four elementary schools of Mexico City, we recorded anthropometry measurements, a fasting blood sample was collected, and metabolic parameters were determined. We obtained information on the foods and beverages that children brought for their school lunch; estimated the caloric and nutritional content; and created food groups to obtain dietary patterns from the energy provided by those groups. Among the 350 schoolchildren (mean age, 7.9 ± 1.2 years) included, 24.9% and 21.7% presented having overweight and obesity, respectively. A total of 89.4% of schoolchildren brought the school lunch from home. Using the K-means method, the following four dietary patterns were identified: (1) sandwiches, *tortas*, and sweetened dairy products were consumed by 13.1% (*n* = 46) of the schoolchildren; (2) sweet snacks were consumed by 50.3% (*n* = 176); (3) sweetened dairy products were brought by 15.1% of the children (*n* = 53); and (4) sandwiches and *tortas* were brought by 21.4% (*n* = 75). These four patterns showed significant differences in terms of the caloric and nutritional contents (*p* < 0.001). Energy sources in the identified patterns were primarily sugars (15.8–40%). No association was found between the anthropometric and metabolic parameters of children and the dietary patterns. No dietary pattern obtained from the school lunch could be considered as healthy, since all of them had high energy content, and a high percentage of the energy was from sugars from ultra-processed foods and beverages.

## 1. Introduction

Overweight and obesity are public health problems that affect children and adults worldwide. These conditions are risk factors for the development of metabolic disorders and chronic noncommunicable diseases from an early age [1]. In Mexico, data from the National Surveys on Health and Nutrition (ENSANUT) from 2012, 2018, and 2020 show that the combined prevalence of overweight and obesity in children aged 5–10 years has remained high: 33.2%, 35.5%, and 38.2%, respectively [2]. This high prevalence is related to family lifestyles characterized by an increase in the consumption of ultra-processed foods, with high amounts of added sugars, low physical activity levels, and an increase of sedentary behaviors [2,3].

The diet of the Mexican population, both in children and in adults, is characterized by low consumption of vegetables, fruits, and wholegrain cereals, and by high consumption of foods high in energy density from added sugars and saturated fats. This is based on the consumption of ultra-processed foods, which are mainly refined flours, sweetened dairy, and non-dairy beverages, among others. The 2018 ENSANUT survey showed that 76.1% of schoolchildren consume snacks and appetizers (salty, fried foods, candy, and desserts); 85.7% of them consume sweetened non-dairy beverages; 44.3% consume fruit; and 43.2% consume legumes on a regular basis [2]. The consumption of foods with high energy content, saturated fats, and abundant refined carbohydrates was positively associated with the risk of overweight or obesity both in children and in adults [4,5].

Food selection, purchase, preparation, and consumption are influenced by availability of foods and ease of access and cost, as well as by the level of food education in the population. Eating patterns are developed early in life; preferences for certain foods and flavors are established early and will remain for the rest of an individual’s life [6]. Parents have great influence on the dietary habits and behavior of children, and they primarily decide the quantity and quality of the foods given to children [7].

In Mexico, the school lunch is comprised of a combination of foods, ready-made meals, and beverages that are consumed while children are at school, regardless of where these products were bought from. These foods should provide the necessary nutrients and energy for this meal, which is eaten during the mid-day break at school [8,9,10]. This meal is generally known as mid-morning snack; however, we have decided to call it school lunch because the results of some studies about schoolchildren living in Mexico City suggest that due to the quantity of energy and macronutrient intake from the food that children eat at school this meal time is a school lunch not a snack meal. Thus, the main mealtimes in Mexico are breakfast, mid-morning snack (*almuerzo*), lunch, and dinner. In Mexico, schoolchildren typically consume their breakfast between 6:30 and 7:30 a.m., school lunch from 10:30 to 11:00 a.m., lunch at home from 2:00 to 3:00 p.m., and dinner at 8:00 p.m. Owing to the long lapse between breakfast and lunch, the school lunch supplies more energy and nutrients than a mid-morning snack [11].

The study of dietary patterns is of interest because foods are consumed in combination, not as individual foods and nutrients [12]. Understanding the nutritional composition of the different meals and the impact that different dietary patterns have on the quality of the diet could help elucidate important relationships between diet and development of diseases [13]. Evaluating dietary patterns instead of the consumption of individual foods provides a wider, more comprehensive view of food consumption in the population. Dietary patterns are defined as nutritional variables that are grouped according to some health and nutrient content criteria, where variables are reduced in number by using statistical techniques [14]. Over the past few decades, Mexico has experienced changes in dietary patterns, going from traditional diets to diets containing more energy-dense food (i.e., foods high in added sugars, refined flours, and saturated fats) [4,5,15]. This has led to unhealthy dietary patterns in children and adults [16,17,18]. A dietary pattern rich in vegetables, fruits, and whole grains could prevent diseases associated with obesity [19]. 

Dietary patterns of Mexican children have been identified by analyzing one-day diets or breakfast foods [5,19,20,21]; however, dietary patterns based on foods prepared at home by parents and eaten at school lunches have not been studied. Moreover, dietary patterns vary among populations due to the differences in food availability, residence area, geographical region, cultural practices, and socioeconomic status, among others. Currently, this issue has become extremely relevant for the implementation of future strategies focused on preventing and reducing the prevalence of overweight and obesity [22,23], or promoting a healthy diet. The objective of this study was to identify patterns of food consumption from the school lunch that children bring from home and its relationship with nutritional status and biochemical indicators.

## 2. Materials and Methods

The present study was cross-sectional, based on information obtained from the basal measurement of a study on an educational intervention for diet and physical activity that targeted parents and school-age children [24]. Students from Grades 1–4 (ages 6–11 years) of both sexes of elementary school were included in the study. They were students from four schools in Mexico City, two private and two public schools, located in the same geographical area, selected by convenience. From the four schools, 817 children were invited to participate; the participation rate was 49.8% (407/817). The low participation rate could be because the original study was a community trial focused on the prevention of overweight and obesity, many parents may not have had the time to participate, or they do not consider overweight and obesity to be health problems yet.

Students were included in the study regardless of their nutritional status (i.e., we included children with normal weight, overweight, or obesity, according to the body mass index (BMI) *z-score)*. Children with any disease or those who were taking any medication that affected the metabolic profile were excluded. Children’s profiles that showed incomplete data (anthropometry measures, school lunch information) were eliminated from the analysis.

The study was approved by the Research, Ethics and Biosafety Committees of the Hospital Infantil de México Federico Gómez (HIM/2013/003), and it was authorized by the Ministry of Public Education and the principals of the participating elementary schools. Before starting the study, the objectives were explained to teachers, parents, and children, and written informed consent and assent were obtained from children and their parents, respectively. 

### 2.1. Measures

#### 2.1.1. Sociodemographic Characteristics

Parents answered a questionnaire, with questions about the educational level of the mother (secondary education or lower, pre-university, professional school, bachelor’s degree, or postgraduate degree), the characteristics of the house, and the goods and services available at home, such as television, refrigerator, washing machine, oven, boiler, land line, computers, Internet access, and automobile; with this information, categories of socioeconomic level were obtained. 

#### 2.1.2. Information on the School Lunch

Before registering the food, each child was told why they would be asked about the food they had in their lunchboxes. The food record was conducted in a place determined by the principals of each school or in the school playground during the school break, the moment when the children consume their food. The survey of the school lunch information was done only once. Qualified nutritionists registered the foods and beverages in the children’s lunchboxes through direct observation; they described foods, ingredients (raw or cooked, type of cooking, and name of the liquid foods), and the estimated amount of each product (g or mL, pieces, portions, tablespoons, teaspoons, cups, and slices, etc.). For processed products, the g and mL described in the packaging were registered. Foods purchased by the caregiver or child before arriving at school, outside of school, or purchased at school during the school break were not included in the analysis. Therefore, if a child did not bring any food or drink from home, they were classified as not bringing school lunch from home and were excluded from this analysis.

A manual of Mexican nutrition projects was used to obtain equivalences of household food measurements and to convert them to g or mL of food and beverages [25]. The amount of each food (in g or mL) included in the school lunch of each child was captured in the software Food Processor SQL (10.11.0 version, 2011, ESHA Research, Salem, Oregon), which contains food composition databases, including information about traditional Mexican foods. For foods not available in the software, the corresponding information was obtained from nutrition tables of Mexican foods [26] or from the nutritional contents stated in the packaging of processed foods and beverages. Subsequently, these data were recorded in the software as well.

We created a database with foods and beverages that made up the school lunch for each child. In this database, information on three standardized recipes generated from the registered foods and beverages was included: (1) sandwiches or *tortas* (white bread or *bolillo*, ham or sausage, cheese, and mayonnaise); (2) Mexican dishes (based on corn with fat); and (3) fresh water with sugar (water with fruit and sugar). The net weight in g of fruits and vegetables was calculated by deducting the weight of the non-edible portion (corresponding to shells and seeds, etc.,) from the total weight of the product [27,28]. Subsequently, the nutritional content of the food was obtained using the Food Processor software. 

Foods and standardized recipes were classified into 24 groups of foods that were mutually exclusive; they were also assigned a group code (Table S1). The composition of macronutrients, the fiber and sugar contents, and other culinary aspects were considered in creating each group. The food groups were as follows: vegetables, fruits, cereals, tubers, ready-to-eat boxed cereal (low in fiber and high in sugar), sweet cookies and cereals, sandwiches/*tortas*, Mexican dishes, cold meat, fast food, fish, eggs, lean meat, legumes, cheese low in fat, cheese high in fat, milk/plain yogurt, sweetened dairy products, oils/fats, seeds, sugary beverages (fresh water with sugar, processed sugary water, carbonated soft drinks, processed juice, and sports drinks), fried products/snacks, and sauces/condiments.

**Figure 1:**
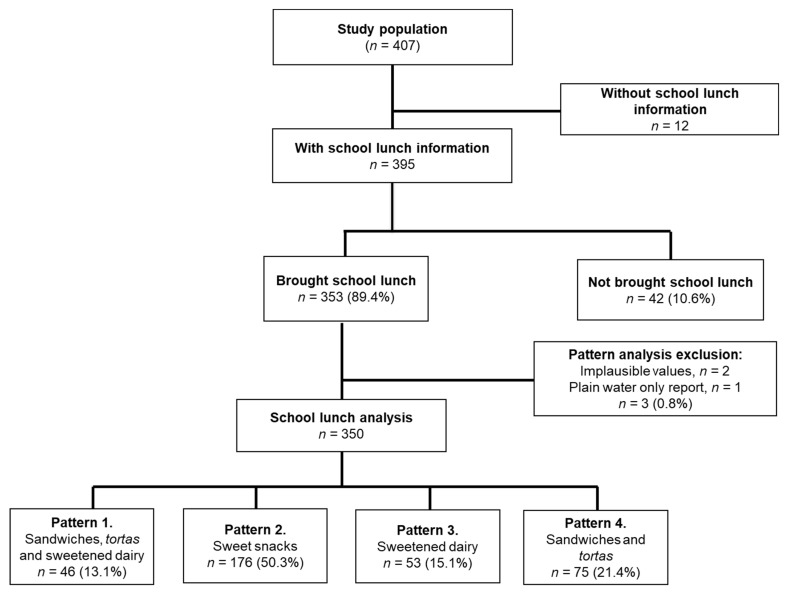
Diagram of the study population.

The percentage of the total energy, proteins, carbohydrates, fats, saturated fats, and simple carbohydrates provided by the school lunch and each of the food groups was obtained. Implausible values were considered when the value was greater or less than 3 SD from the mean of calories consumed at school lunch; therefore, they were excluded from the analysis (*n* = 2) (Figure 1).

#### 2.1.3. Anthropometric Measurements

Height was measured with a stadiometer (SECA model-225, SECA Corp., Hamburg, Germany), with an accuracy of 0.1 cm. Weight was measured with a digital scale (SECA model-882, SECA Corp., Hamburg, Germany), with an accuracy of 0.1 kg, and the waist circumference was measured with a flexible, non-elastic measuring tape (SECA model-200). The measurements were made by two previously standardized nutritionists following international procedures [29]. Children were measured without shoes while wearing light clothes. They were instructed to stand in the center of the platform from the scale or stadiometer, with arms dangling by the sides of the torso and gazing in the Frankfort plane.

The BMI *z-score* was calculated with the weight, height, age, and sex of the child. Using World Health Organization data from 2007 as a reference, children were classified as having low weight (*z-score* < −2), normal weight (*z-score* of −2 to <1), overweight (*z-score* of ≥1 to <2), and obesity (*z-score* of ≥2) [30]. Waist circumference was used as proxy to evaluate the central visceral adiposity [31]; the waist circumference percentile was calculated by taking into account the age, sex, and height of the child and using the waist circumference tables for Mexican children [32]. Children were classified into three groups according to their waist circumference percentile (pc) as follows: normal (pc < 75th), at high risk (pc 75th to 89th), and at very high risk (pc ≥ 90th).

#### 2.1.4. Biochemical Determinations

A sample of venous blood (6 mL) was collected from the children after 12 h of fastening, after taking the blood sample, the children were offered breakfast. Samples were centrifuged at 2000 rpm for 10 min at 4 °C, then serum was obtained. Serum aliquots were then sent to the Central Laboratory of the Hospital Infantil de México for determination of the following metabolic parameters: glucose, triglycerides, total cholesterol (C-total), and cholesterol of high-density lipoproteins (HDL-C) (ILAB 300, Instrumentation Laboratory, Barcelona, Spain). LDL-C levels were calculated using Friedewald equation [33]. Insulin was determined via a chemiluminescence assay (IMMULITE 2000, Euro, DPC, Llanberis, UK). HOMA-IR was obtained from the following equation: [fasting glucose (mg/dL) × fasting insulin (μU/mL)/405] [34].

### 2.2. Statistical Analysis

The information on the characteristics of the dwelling and the goods and services available in the home was analyzed through principal components analysis; the score obtained was divided in tertiles to classify the socioeconomic status of study sample into low, medium, and high.

Measures of central tendency were used to describe basal characteristics of the study population. Means and standard deviations were obtained for continuous variables with normal distribution, while medians and interquartile range (IQR) were calculated for continuous variables without normal distribution, and percentages for categorical variables. Children’s height and weight were adjusted by age and sex.

The percentage of energy that each of the 24 groups of foods provided to the total energy was calculated by using the following formula: ∑ (energy of each food from the same group × 100/total energy of the school lunch). The percentage of energy of each group of foods was used in the cluster analysis, in which a K-means method was used to obtain maximum homogeneity and the greatest difference among groups. Three to five solutions were tested to maximize the Euclidean distance among groups; a solution of four dietary patterns was chosen, arranged by their size and easiness to be interpreted in relation to diet. We also calculated the energy and grams of macronutrients that each dietary pattern provided per 100 g. 

In addition, Kruskal–Wallis and Pearson’s chi-squared tests were used to compare the contents of energy, macronutrients, and the percentage of children bringing plain water between the dietary patterns identified. If the results of the Kruskal–Wallis test were statistically significant, then Dunn’s test with a Bonferroni correction post hoc was used to determine exactly which dietary patterns were identified as statistical differences. To compare the socioeconomic and anthropometric characteristics and the biochemical parameters of the children according to the identified dietary pattern, Pearson’s chi-squared test was used for categorical variables and ANOVA and Kruskal–Wallis test for continuous variables based on data distribution.

Values of *p* < 0.05 were considered statistically significant for all analyses. The analysis was carried out with Stata v17.0 (StataCorp, College Station, TX, USA).

## 3. Results

A total of 350 schoolchildren with a mean age of 7.9 ± 1.2 years were included in the analysis, 45.4% of whom were females. Table 1 shows the socioeconomic, anthropometric, and metabolic characteristics of the schoolchildren; 24.9% had overweight and 21.7% had obesity. 

**Figure 2:**
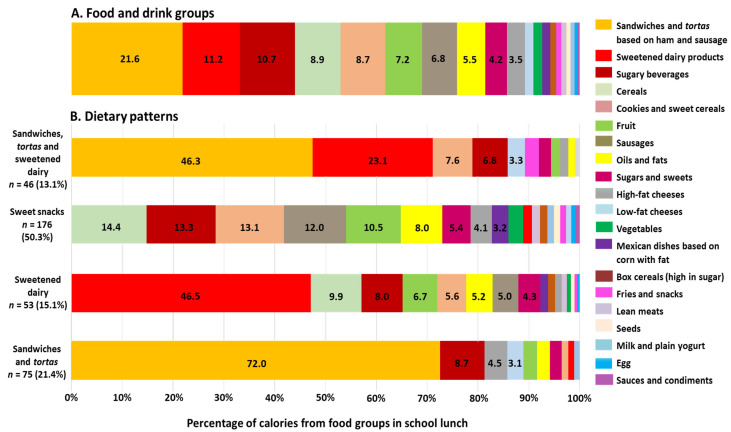
(**A**) Energy percentage provided by the different food groups included in the school lunch. (**B**) Dietary patterns from school lunch consumed by children (*n* = 350) and energy percentage provided by the food groups.

Figure 2A shows the energy of each group of foods and beverages included in the school lunch (Table S1). The highest percentages came from food groups such as sandwiches and *tortas* (21.6%), sweetened dairy products (11.2%), and sweetened beverages (10.7%).

Four dietary patterns were obtained from the school lunch (Figure 2B and Table S2): (1) The pattern of sandwiches, *tortas*, and sweetened dairy products, consumed by 13.1% (*n* = 46) of schoolchildren, was characterized by the high contribution of energy from sandwiches, *tortas*, sweetened dairy products, sweet cookies and cereals, and sweetened beverages. (2) The sweet snacks pattern, consumed by 50.3% (*n* = 176) of the schoolchildren, was characterized by a great variety of foods and beverages; high energy intake coming from sweetened drinks, cookies, sweet cereals, and confectionery (31.8%) was observed. Further, the highest caloric content came from fruit (10.5%). (3) The pattern of sweetened dairy products, consumed by 15.1% (*n* = 53) of schoolchildren, was characterized by sweetened dairy products, sweetened drinks, fruits, cookies, and sweet cereals, which provided a high amount of energy to this pattern. (4) The pattern of sandwiches and *tortas*, consumed by 21.4% (*n* = 75) of the schoolchildren, was mainly characterized by consumption of sandwiches, *tortas*, and sweetened drinks. 

Table 2 describes the energy and macronutrient contribution according to the dietary patterns. The median of the energy intake from the schoolchildren’s lunch was 448 kcal (IQR: 324–581); 54.2% of the energy intake came from carbohydrates (24.7% from simple carbohydrates), 12.1% from proteins, and 34.6% from fats (10.5% from saturated fats). Fiber content was 2.9 g (IQR: 1.6–4.9). The school lunch of 41.7% of children included plain water.

Caloric and macronutrient intake differed among the identified dietary patterns (*p* < 0.001). The dietary pattern of sandwiches, *tortas*, and sweetened dairy products had the highest caloric content (657 kcal; IQR: 575–725) and, proportionally, the lowest consumption of plain water (28.3%). The sweet snacks pattern was the one with the lowest caloric intake (369 kcal; IQR: 255–504) and the lowest percentage of calories came from proteins and saturated fats. The pattern of sweetened dairy products showed the highest percentage of energy from carbohydrates (59.1%, from which 39.8% came from simple carbohydrates); however, the lowest percentage of energy came from fats (27.6%). Finally, the pattern of sandwiches and *tortas* showed the lowest energy intake from carbohydrates (45.9%, from which 15.8% came from simple carbohydrates), but the highest energy came from fats (39.8%, from which 12.3% came from saturated fats). This pattern also showed the highest percentage of children bringing plain water (46.7%). When calculating the energy content and the g of macronutrients per 100 g for each of the dietary patterns, the one with sandwiches, *tortas*, and sweetened dairy products showed the highest energy density, whereas the sweet snacks pattern showed the lowest energy content. Notably, both the pattern of sandwiches, *tortas*, and sweetened dairy products and the pattern of sweetened dairy products were the ones that showed the highest simple carbohydrates content; both patterns are characterized by including ultra-processed drinks.

Table 3 and Table 4 describe anthropometric and biochemical parameters according to dietary patterns. No significant differences were observed among dietary patterns.

## 4. Discussion

Mexico has some of the highest prevalence of overweight and obesity in children and adults in the world [2,35]; therefore, describing the dietary patterns of schoolchildren by studying the school lunch that they bring from home acquires great relevance for the implementation of future intervention strategies. Our results show that none of the dietary patterns observed from the school lunch was healthy. All of them include a great consumption of foods and beverages high in added sugars. These were mainly ultra-processed products, the consumption of which entails a risk of developing overweight or obesity [19]. The patterns identified provided between 15.8% and 40% of the energy from simple carbohydrates, which exceeds the recommendation about the daily energy intake from sugars (10%) [36].

Dietary environment influences the consumption decisions of the population, mainly through the availability and access to different types of foods and beverages where people live, study, work, and perform their daily activities. Children are a vulnerable population group because they are at an age in which they acquire enjoyment and preference for tastes, and they are more likely to believe and repeat what they observe and hear around them. Foods chosen for family consumption should be a healthy option for children, which is why it is important for families to have some nutritional guidance in making good choices and in planning their diet [6]. This is an important area of attention since the characteristics of the school lunch found in this study point to unhealthy dietary habits within the household.

Our results agree with those shown in the Survey on Health and Nutrition in Mexico from 2018 [37], which reported a high consumption of non-recommended foods in schoolchildren, such as sweetened non-dairy drinks (89%), Mexican dishes, confectionery and desserts (70.7%), sweet cereals (47.3%), sweetened dairy drinks (32.1%), and processed meat (6%). These dietary choices represent a combination of industrialized foods and preparations, characterized by a high energy density and a high amount of lipids, saturated fats, and added sugars.

Similar to our study, this high consumption of sugars in the dietary patterns of Mexican schoolchildren has consistently been reported by other authors. Zamora Gasga, et al. [21] identified three types of diet in this age group: “Mexican and modified,” “Mexican and traditional,” and “Mexican and alternative”; within these patterns, added sugars, confectionery, desserts, and sugary drinks were included. Afeiche MC, et al. [20] identified six dietary patterns from the information on breakfast: milk and sweet rolls, *tortillas* and beans, sweetened beverages, sandwiches and quesadillas, eggs, and cereals with milk. These products showed both traditional and Westernized dietary patterns. In all of them, the consumption of sweetened beverages was identified with sugar and corn syrup (high in fructose). In a study of U.S. children, the foods that schoolchildren brought to school were characterized according to the level of processing. They found that 70% of the caloric content of food brought from home to school was highly processed. Foods categorized as snack foods and desserts contributed the greatest percentage of energy to the highly processed category [38]. On the other hand, in a study of Canadian children, on average, foods reported at lunch provided 25.8% of daily calories on school days (between 451 and 584 kcal, depending on eating location). Furthermore, lunch on school days contributed to proportionally lower intakes of many healthful foods such as dark green and orange vegetables, whole fruit, whole grains, and fluid milk [39]. 

In Mexico, the consumption of very high levels of added sugar has been reported in the past; this is more than three times the amount recommended by the World Health Organization. Beverages are the greatest source of sugar in the diet of most of the children, teenagers, and young adults [40,41]. The high consumption of sweetened beverages must receive critical attention, so that the population does not get used to an excessively sweet taste from an early age. In this study, we observed that only 41.7% of children brought plain water in their lunchboxes; in contrast, all patterns showed a consumption of sweetened drinks as follows: fruit-infused water with added sugar, processed sweet water, carbonated soft drinks, processed juice, sports drinks, and sweetened dairy products. Sweetened dairy products such as milk and yogurt have advertising oriented toward children. These drinks provide a large amount of energy due to the high amount of added sugar, so it is recommended that children include milk in their school lunch, but without added sugar or flavorings. Families are encouraged to have dairy products such as plain yogurt or milk sent with the school lunch, with the option of adding fresh fruit for flavor. This is an opportunity to present interventions that promote the consumption of plain water and dairy beverages with no added sugar.

In Mexico, the dietary patterns characterized by a high intake of legumes and snacks (popcorn, pumpkin seeds, and peanuts) and a low intake of sugary drinks have been associated with lower weight and BMI [21]. The dietary patterns that include sweet cereals and corn-based dishes and those Western patterns characterized by the consumption of sweetened beverages, fried foods, pastries, and sweet cereals have been associated with higher overweight and obesity levels when compared to a rural pattern characterized by the consumption of *tortillas* and legumes [19]. Although we did not find associations between dietary patterns identified at school lunch and anthropometric parameters, it is not possible to rule out that the characteristics of these unhealthy patterns spread to other mealtimes at home, since the school lunches analyzed come from the homes of schoolchildren.

Different studies point out the negative effects of ultra-processed foods, which are high in sugars and fats, in children’s health: increase of total cholesterol, low-density lipoproteins (C-LDL) [42] triglycerides, blood pressure, glucose, insulin [43,44], and waist circumference [45]. Furthermore, these were associated with a higher risk of cardiometabolic disease [46,47,48]. Although in our study we did not identify an association between the metabolic profile and dietary patterns from the school lunch, it is important to highlight that, when highly processed foods with high energy intake are consumed on a regular basis, there will invariably be a high risk of developing cardiometabolic disorders in the future. It is essential for parents to have more information about the nutritional quality of foods, allowing them to choose, buy, and prepare healthy food.

We observed little inclusion of foods such as fruits and vegetables, fish, legumes, lean meat, eggs, sugar-free dairy products, and seeds. This study shows the need for nutritional guidance for parents, schoolchildren, and the entire educational community, emphasizing the recommendation of consuming foods that provide proteins, unsaturated fats, and fiber. A healthy school lunch must include a dish based on wholegrain cereals, a portion of a source of proteins, a piece of fruit and/or vegetable, and plain water [8]. Fruits and vegetables must be a part of the school lunch on a daily basis; this meal is an opportunity for children to consume this group of foods and to meet their vitamin, mineral, and fiber needs. 

In this study, two of the identified dietary patterns included sandwiches and *tortas*, which are preparations made with white bread or rolls, ham or sausages, fresh cheese, and mayonnaise. These foods provide between 46.3% and 72% of the total energy, and up to 12% of the energy comes from cold meat mainly ham—products high in saturated fats, sodium, nitrates, or nitrites, substances that entail health risks [49].

The recommended percentage of energy that each meal should provide are 25% for breakfast, 30% for lunch, 15% for dinner, and two light meals (including the school lunch of 15% each. According to this recommendation, the school lunch should provide approximately 237 kcal (225–249 kcal) as this meal time has been considered as a collation [8,9,10]. However, this study showed that the energy intake of the school lunch was higher in all the dietary patterns (an average of 448 kcal, IQR: 324–581), as the pattern of sandwiches, *tortas*, and sweetened dairy products was the one with the highest caloric content [657 kcal, (IQR: 575–725)]. These results are consistent with the information reported by other studies conducted in Mexico [11,50]. In the study by Martínez et al., the children gave information about the foods that they consumed five days a week during school hours (inside the school and on the way to and from school and home); the median calorie intake per day was 515 kcal (IQR:366–693) [50]. The high energy consumed in the school lunch could mean that the school lunch tends to provide high energy intake compared to what is expected from light, intermediate meals or snacks in complex places such as Mexico City, where commute to school or work is longer and the period of time between breakfast and lunch at home is longer as well. In addition, preliminary observations point out that a significant amount of children do not have breakfast and those who have it do not eat foods in the proper amount or quality [20]. Taking these observations into account, alternatives must be considered for distributing the caloric intake among the various meals. The convenience of decreasing the energy content at breakfast and dinner when the school lunch provides 20–25% of the caloric intake must also be considered. Finally, it is necessary to know if children have or do not have the afternoon snack.

The dietary patterns from the school lunch identified in this study, show the intake of a high percentage of calories from sweetened foods as sweetened milk, sugar beverages, cereals, and cookies. The quality of those dietary patterns could be improved by omitting sweetened foods and reducing portion sizes of foods consumed. Because overweight and obesity represent health problems in Mexican schoolchildren, actions to prevent and control these problems are required; they should go beyond the individual level and involve the household environment, for example, implement community interventions that help children and parents to select and prepare food for school lunch in advance. It is necessary that the nutritional recommendations and guidelines given by health and education authorities be guided by knowledge of the population’s eating habits, so that the recommendations are culturally acceptable to enable a change in the quality of school lunch. Improved school food policies and appropriate, nutritionally adequate guidance for planning school lunch menus are needed. In addition, legislation on the quality of foods provided by the food industry is required. 

The limitation of this study is that the evaluation was of only one meal, which may or may not be representative of the consumption through the rest of the day, future studies are needed to evaluate the diet of one or more days of schoolchildren to know the energy distribution of each meal time. This information could be useful to give more culturally acceptable nutritional recommendations on the distribution of total energy from the different meals in this population. In addition, in this study foods bought from inside or outside the school were not taken into account; these foods could increase the energy consumption. Likewise, the children’s level of physical activity was not included; this information could explain the lack of association between anthropometric and biochemical data and the identified dietary patterns. 

## 5. Conclusions

No dietary pattern obtained from the school lunch that was brought from home was identified as healthy; all of them had a high energy content, with a higher percentage of energy from sugars, which came mostly from ultra-processed foods and drinks. 

Identifying, describing, and understanding dietary patterns that schoolchildren consume from the school lunch that comes from home acquires great relevance for implementing future strategies where nutritional guidance will be designed for parents and children so that they can make adequate choices and preparation of foods.

## Figures and Tables

**Table 1:** Characteristics of the study population.

Characteristics	*n* = 350
Age (years) (mean ± SD)	7.9 ± 1.2
Sex (female), *n* (%)	159 (45.4)
Weight (kg) ^†^ (mean ± SD)	29.1 ± 5.0
Height (cm) ^†^ (mean ± SD)	126.1 ± 7.3
BMI *z-score* (mean ± SD)	0.90 ± 1.34
BMI classification, *n* (%)	
Normal (−2 < *z-score* < 1)	187 (53.4)
Overweight (1 ≤ *z-score* < 2)	87 (24.9)
Obesity (*z-score* ≥ 2)	76 (21.7)
Waist circumference (percentile) (mean ± SD)	52 ± 21.3
Metabolic parameters (*n* = 278) ^a^ (median-IQR)	
Glucose (mg/dL)	87 (81–91)
Cholesterol (mg/dL)	166 (147–183)
Triglycerides (mg/dL)	70 (51–97)
C-HDL (mg/dL)	49 (40–57)
C-LDL (mg/dL)	98 (84–115)
Insulin (μU/mL)	3.2 (2–6)
HOMA-IR *	0.68 (0.44–1.29)
Educational level of the mother (*n* = 323) ^b^, *n* (%)	
Secondary or lower	52 (16.1)
Pre-university or technical school	129 (39.9)
Bachelor’s Degree or Postgraduate Degree	142 (44.0)
Socioeconomic level (*n* = 311) ^c^, *n* (%)	
Low	88 (28.3)
Medium	117 (37.6)
High	106 (34.1)

^†^ Adjusted for age and sex by multiple linear regression. BMI: body mass index; RIC: interquartile range. * HOMA: [fasting glucose (mg/dL) × fasting insulin (μU/mL)/405]. ^a^ Missing data for 72 children. ^b^ Missing data for 27 children. ^c^ Missing data for 39 children.

**Table 2:** Energy and macronutrient contribution by dietary patterns.

Energy and Macronutrients	All *n* = 350	Sandwiches, *Tortas*, and Sweetened Dairy Products *n* = 46 (13.1%)	Sweet Snacks *n* = 176 (50.3%)	Sweetened Dairy Products *n* = 53 (15.1%)	Sandwiches and *Tortas* *n* = 75 (21.4%)	*p* Value
	**Median (IQR)**					
Energy (kcal)	448 (324–581)	657 (575–725) ^1,2,3^	369 (255–504) ^5^	471 (287–572)	468 (401–542)	<0.001
Carbohydrates (g)	58.9 (37.8–79.6)	85.8 (79–102) ^1,2,3^	48.4 (27.7–69.2) ^4^	62.8 (49.2–83.8)	54.8 (42.8–65.8)	<0.001
Carbohydrates (%)	54.2 (44.4–64.3)	54.7 (50.2–60.1) ^3^	54.6 (40.4–68.4) ^4,5^	59.1 (49.3–73.1) ^6^	45.9 (41.1–53.6)	<0.001
Proteins (g)	14.4 (8.3–19.5)	21.0 (19.1–23.5) ^1,2,3^	10.6 (5.7–16.4) ^5^	13.4 (8.2–20.4)	15.1 (12.9–18.1)	<0.001
Proteins (%)	12.1 (9.5–14.8)	13.2 (11.5–14.8) ^1^	10.7 (7.7–14.8)	11.1 (9.8–14.2) ^6^	13.5 (11.7–14.9)	<0.001
Fats (g)	16.7 (11.2–25.7)	24.4 (19.5–28.8) ^1,2,3^	15.1 (6.7–23.9) ^5^	14.7 (5.8–26.1) ^6^	19.9 (14.0–26.2)	<0.001
Fats (%)	34.6 (26.5–43.6)	33.1 (28.4–39.3) ^3^	35.0 (21.2–45.8) ^4,5^	27.6 (17.5–35.9) ^6^	39.8 (33.4–45.5)	<0.001
Simple carbohydrates (g)	27.8 (11.5–45)	46.9 (30.8–54.6) ^1,3^	21.4 (9.9–39.1) ^4^	45.7 (31.1–56.3) ^6^	18.8 (6.2–28.9)	<0.001
Simple carbohydrates (%)	24.7 (13.2–38.8)	27.7 (20.0–33.8) ^2,3^	26.2 (12.6–42.8) ^4,5^	39.8 (27.7–55.3) ^6^	15.8 (5.9–23.5)	<0.001
Saturated fats (g)	5.8 (2.9–8.3)	7.7 (6.0–9.1) ^1,2^	3.8 (1.7–7.1) ^5^	5.6 (2.0–9.2)	6.4 (4.3–8.5)	<0.001
Saturated fats (%)	10.5 (6.6–14.8)	10.8 (8.9–12.7)	9.4 (4.0–15.4) ^5^	10.5 (6.1–13.4) ^6^	12.3 (9.8–15.4)	<0.001
Fiber (g)	2.9 (1.6–4.9)	4.4 (3.2–5.3) ^1,3^	2.5 (1.3–4.9) ^4^	4.3 (3.2–6.0) ^6^	2.1 (1.6–3.3)	0.003
Bringing plain water, *n* (%)	146 (41.7)	13 (28.3)	77 (43.8)	21 (39.6)	35 (46.7)	0.206
	**Content in 100 g**					
Energy (kcal)	80 (50–110)	111 (92–134) ^1,2,3^	70 (43–106)	80 (43–102)	80 (65–100)	<0.001
Carbohydrates (g)	10.6 (6.3–14.5)	15.5 (12.5–18.2) ^1,2,3^	9.4 (5.1–13.2)	10.6 (6.9–14.6)	10.3 (6.4–13.5)	<0.001
Proteins (g)	2.4 (1.4–3.5)	3.6 (2.5–4.6) ^1,2,3^	1.8 (1.0–3.1) ^5^	2.2 (1.3–3.2)	2.6 (2.1–3.5)	<0.001
Fats (g)	3.1 (1.8–4.8)	4.3 (3.1–5.3) ^1,2,3^	2.6 (1.3–4.8) ^5^	2.4 (1.0–4.2) ^6^	3.5 (2.4–4.5)	<0.001
Simple carbohydrates (g)	4.9 (2.4–7.7)	7.6 (5.2–9.2) ^1,3^	4.2 (2.0–7.0) ^4^	7.2 (4.6–9.8) ^6^	3.8 (1.0–5.5)	<0.001
Saturated fats (g)	1.0 (0.4–1.5)	1.2 (1.0–1.7) ^1,2^	0.7 (0.3–1.5) ^5^	0.7 (0.4–1.5) ^6^	1.1 (0.8–1.5)	<0.001
Fiber (g)	0.5 (0.3–0.8)	0.7 (0.5–1.0) ^1,3^	0.5 (0.2–0.9) ^4^	0.7 (0.4–0.9) ^6^	0.4 (0.5–0.6)	<0.001

Kruskal–Wallis test, Pearson’s chi-squared test. Dunn’s test of multiple comparisons, ^1^ Sandwiches, *tortas*, and sweetened dairy products vs. sweet snacks: *p* < 0.05; ^2^ sandwiches, *tortas*, and sweetened dairy products vs. sweetened dairy products: *p* < 0.05; ^3^ sandwiches, *tortas*, and sweetened dairy products vs. sandwiches and *tortas*: *p* < 0.05; ^4^ Sweet snacks vs. sweetened dairy products: *p* < 0.05; ^5^ sweet snacks vs. sandwiches and *tortas*: *p* < 0.05; ^6^ sweetened dairy products vs. sandwiches and *tortas*: *p* < 0.05.

**Table 3:** Socioeconomic, anthropometric, and clinical characteristics according to dietary patterns.

	Sandwiches, *Tortas*, and Sweetened Dairy Products *n* = 46 (13.1%)	Sweet Snacks *n* = 176 (50.3%)	Sweetened Dairy Products *n* = 53 (15.1%)	Sandwiches and *Tortas* *n* = 75 (21.4%)	*p* Value *
Age (years), (mean ± SD)	7.7 ± 1.1	7.9 ± 1.2	8.0 ± 1.2	7.8 ± 1.2	0.594
Sex (female), *n* (%)	20 (43.5)	87 (49.4)	22 (41.5)	30 (40.0)	0.488
BMI *z-score* (mean ± SD)	0.87 ± 1.3	0.90 ± 1.4	0.91 ± 1.5	0.92 ± 1.2	0.997
BMI *z-score* classification, *n* (%)					
Normal (−2 < *z-score* < 1)	25 (54.4)	91 (51.7)	26 (49.1)	45 (60.0)	
Overweight (1 ≤ *z-score* < 2)	13 (28.2)	43 (24.4)	14 (26.4)	17 (22.7)	
Obesity (*z-score* ≥ 2)	8 (17.4)	42 (23.9)	13 (24.5)	13 (17.3)	0.815
WC (percentile) (mean ± SD)	50.1 ± 19.8	53.8 ± 21.5	52.1 ± 20.6	49.4 ± 21.6	0.429
WC percentile classification, *n* (%)					
Normal (percentile < 75th)	41 (89.1)	139 (79.9)	46 (86.8)	64 (85.3)	
High risk (percentile 75th to 89th)	3 (6.5)	23 (13.2)	4 (7.5)	8 (10.7)	
Very high risk (percentile ≥ 90th)	2 (4.4)	12 (6.9)	3 (5.7)	3 (4.0)	0.715
Educational level of the mother, *n* (%)					
Secondary or lower	6 (14.0)	28 (17.5)	5 (10.0)	13 (18.6)	
Pre-university or technical	18 (41.9)	59 (36.9)	27 (54.0)	25 (35.7)	
Bachelor’s degree or postgraduate degree	19 (44.2)	73 (45.6)	51 (36.0)	32 (45.7)	0.444
Socioeconomic level, *n* (%)					
Low	16 (30.8)	11 (26.8)	36 (33.3)	25 (23.2)	
Medium	21 (40.3)	22 (53.7)	33 (30.6)	40 (37.0)	
High	15 (28.9)	8 (19.5)	39 (36.1)	43 (39.8)	0.281

* Kruskal–Wallis test, Pearson’s chi-square test or ANOVA. WC: waist circumference

**Table 4:** Metabolic parameters according to dietary patterns.

Biochemical Parameters	Sandwiches, *Tortas*, and Sweetened Dairy Products *n* = 33	Sweet Snacks *n* = 149	Sweetened Dairy Products *n* = 37	Sandwiches and *Tortas* *n* = 59	*p * Value *
	Median (IQR)				
Glucose (mg/dL)	87 (81–93)	86 (81–90)	86 (79–92)	87 (81–90)	0.700
Cholesterol (mg/dL)	168 (155–180)	164 (148–184)	168 (142–185)	164 (147–178)	0.918
Triglycerides (mg/dL)	73 (55–101)	72 (55–99)	66 (53–98)	67 (49–88)	0.515
C-HDL (mg/dL)	52 (43–54)	50 (40–58)	45 (41–52)	49 (39–57)	0.342
C-LDL (mg/dL)	103 (88–110)	95 (81–115)	105 (84–119)	98 (84–120)	0.570
Insulin (μU/mL)	3.8 (2.2–6.7)	3.3 (2.0–6.9)	2.6 (1.9–5.2)	2.8 (1.9–5.3)	0.075
HOMA-IR	0.83 (0.47–1.54)	0.69 (0.45–1.52)	0.55 (0.42–1.17)	0.55 (0.43–1.05)	0.150

* Kruskal–Wallis test. IQR: interquartile range.

## Data Availability

The data presented in this study are available on request from the corresponding author. The data are not available publicly.

## Supplementary Materials

The following supporting information can be downloaded at: https://www.mdpi.com/article/10.3390/ijerph191811650/s1. Table S1: Groups of foods. Table S2: Children’s dietary patterns from the school lunch.
