# Supplementary material for: Evidence of Unhealthy Dietary Patterns in the School Lunch Sent from Home for Children in Mexico City
Source: Int J Environ Res Public Health. 2022 Sep 15;19(18):11650. doi: 10.3390/ijerph191811650 (PMC9517523; doi:10.3390/ijerph191811650)
Supplement: Supplementary file 1 [file ijerph-19-11650-s001.zip › ijerph-1815648-supplementary.pdf]

Table S1. Groups of foods

Group code	% Energy in school lunch	Food groups	Foods
1	1.7	Vegetables	Carrots, tomatoes, lettuce, cucumbers, and Mexican yams
2	7.2	Fruits	Bananas, apples, pears, kiwis, guavas, grapes, peaches, figs, tangerines, oranges, pineapples, and watermelons
3	8.9	Cereals	Rice, spaghetti, pasta soup, breadcrumbs, flour, crackers, and bread
4	0.1	Tubers	Home-made potatoes
5	1.1	Ready-to eat cereal boxes (low in fiber, high in sugar)	Corn flakes, puffed rice, and Froot loops
6	8.7	Sweet cookies and cereals	Sweet cookies, coated or stuffed, sweetened cereal bars, rice with milk, *churros*, hot cakes, waffles, and cake.
7	21.6	Sandwiches and *tortas* made from ham and sausages	Sandwiches and *tortas* whose main ingredients are ham and sausages
8	1.6	Mexican dishes based on corn with fat	*Quesadillas*, *tlacoyos* (thick *tortilla* filled with beans or other ingredients), tamales, and *tacos dorados* (golden crispy tacos)
9	6.8	Cold meat	Ham, *chorizo*, sausages, bacon, and nuggets (not included in the sandwich or *torta*).
10	0.3	Fast food	Pizza, Kentucky Fried Chicken®.
11	0.2	Fish	Tuna, fish fillet
12	0.5	Eggs	Whole eggs
13	1.0	Lean meat	Chicken breasts, beef, and pork
14	0.2	Legumes	Beans
15	1.7	Cheese low in fat	Fresh cheese
16	3.5	Cheese high in fat	Manchego cheese (made from sheep's milk), American cheese, cream cheese, and goat cheese
17	0.8	Milk and plain yogurt	Non-flavored, sugar-free milk, and plain yogurt
18	11.2	Sweetened dairy products	Flavored milk and yogurt, sweetened and fermented dairy products
19	5.5	Oils and fats	Oil, avocado, butter, mayonnaise, and cream
20	0.8	Seeds	Nuts, almonds, plain peanuts, pumpkin seeds, and sesame seeds
21	10.7	Sugary beverages	Fresh water with sugar, processed sugary water, carbonated soft drinks, processed juice, and sports drinks
22	4.2	Confectionery	Table sugar, honey, chocolate bars, jam, jelly fruit candy, candy, flavored jelly, crème caramel, fruit in syrup, condensed milk, hazelnut cream, and peanut butter
23	1.0	Fried products and snacks	Chips, popcorn, fried products based on corn or flour
24	0.5	Sauces and condiments	Ketchup, *chipotle*, chile powder, salt, spicy sauce, and soy sauce

Table S2. Children's dietary patterns from the school lunch

**Sandwiches, *tortas*, and sweetened dairy products** *n* = 46 (13.1%)		**Sweet snack** *n* = 176 (50.3%)		**Sweetened dairy products** *n* = 53 (15.1%)		**Sandwiches and *tortas*** *n* = 75 (21.4%)	
**Food group**	**% Calories**	**Food group**	**% Calories**	**Food group**	**% Calories**	**Food group**	**% Calories**
Sandwiches and *tortas*	46.3	Cereals (without added sugar)	14.4	Sweetened dairy products	46.8	Sandwiches and *tortas*	72.0
Sweetened dairy products	23.1	Sugary beverages	13.3	Cereals (without added sugar)	9.9	Sugary beverages	8.7
Sweet cereals and cookies	7.6	Sweet cereals and cookies	13.1	Sugary beverages	8.0	Cheese high in fat	4.5
Sugary beverages	6.8	Cold meat	12.0	Fruits	6.7	Cheese low in fat	3.1
Cheese low in fat	3.3	Fruits	10.5	Sweet cereals and cookies	5.6	Fruits	2.7
Fried products and snacks	2.7	Oils and fats	8.0	Oils and fats	5.3	Oils and fats	2.5
Confectionery	2.3	Confectionery	5.4	Cold meat	5.0	Confectionery	2.3
Fruits	1.8	Cheese high in fat	4.1	Confectionery	4.3	Sweet cereals and cookies	1.3
Cheese high in fat	1.5	Mexican dishes based on corn with fat	3.2	Ready-to eat cereal boxes (high in sugar)	1.4	Sweetened dairy products	1.1
Oils and fats	1.3	Vegetables	2.8	Cheese high in fat	1.3	Fast food	0.4
Cereals (without added sugar)	0.9	Sweetened dairy products	1.7	Lean meat	1.0	Vegetables	0.3
Ready-to eat cereal boxes (high in sugar)	0.7	Lean meat	1.6	Vegetables	0.8	Lean meat	0.3
Seeds	0.4	Ready-to eat cereal boxes (high in sugar)	1.4	Seeds	0.7	Ready-to eat cereal boxes (high in sugar)	0.2
Vegetables	0.3	Milk and plain yogurt	1.3	Milk and plain yogurt	0.6	Eggs	0.1
Milk and plain yogurt	0.3	Seeds	1.3	Fried products and snacks	0.5	Legumes	0.1
		Fried products and snacks	1.1	Eggs	0.5		
		Cheese low in fat	1.0	Fish	0.3		
		Sauces and condiments	0.8	Sauces and condiments	0.3		

	Eggs	0.8	Legumes	0.2
	Fish	0.4	Tubers	0.1
	Fast food	0.4		
	Legumes	0.3		
	Tubers	0.2		
